# Retinal Characteristics in Eyes With Retinal Vein Occlusion Using Widefield Swept-Source Optical Coherence Tomography Angiography

**DOI:** 10.1167/iovs.67.3.45

**Published:** 2026-03-19

**Authors:** Johannes Iby, Judith Kreminger, Heiko Stino, Michael Niederleithner, Lusine Yeghiazaryan, Thomas Schlegl, Wolfgang Drexler, Tilman Schmoll, Rainer A. Leitgeb, Andreas Pollreisz, Ursula Schmidt-Erfurth, Stefan Sacu

**Affiliations:** 1 Department of Ophthalmology and Optometry, Medical University of Vienna, Vienna, Austria; 2 Vienna Clinical Trial Center (VTC), Department of Ophthalmology and Optometry, Medical University of Vienna, Vienna, Austria; 3 Center for Medical Physics and Biomedical Engineering, Medical University of Vienna, Vienna, Austria; 4 Institute of Medical Statistics, Center for Medical Data Science, Medical University of Vienna, Vienna, Austria; 5 Carl Zeiss Meditec, Dublin, California, United States

**Keywords:** retinal vein occlusion (RVO), central retinal vein occlusion (CRVO), branch retinal vein occlusion (BRVO), widefield optical coherence tomography angiography (WF-OCTA), color fundus (CF) imaging

## Abstract

**Purpose:**

The purpose of this study was to investigate vascular alterations and their association with non-perfusion areas (NPAs) in eyes with retinal vein occlusion (RVO) using custom built widefield swept-source optical coherence tomography angiography (WF-OCTA) and ultra-WF color fundus photography (UWF-CF).

**Methods:**

Patients with RVO and symptoms >3 months underwent single-capture 65 degrees WF-OCTA imaging using a custom-built prototype (A-scan rate: 1.7 megahertz [MHz]). Additionally, UWF-CF images were acquired. Retinal features, including collateral vessels (CVs), intraretinal/preretinal hemorrhage (IRH), intraretinal fluid (IRF), microaneurysms, crossing-signs (CS), neovascularization, ghost-vessels (GVs), exudates, foveal avascular zone (FAZ), and NPAs were graded on an extended Early Treatment Diabetic Retinopathy Study (ETDRS) grid up to 18 mm. NPAs were annotated manually on en face images using Fiji. The presence of retinal findings was then correlated with NPA size.

**Results:**

Eighty-one eyes (48 right eyes and 33 left eyes) of 81 patients (women = 50.6%, mean age = 65 ± 12.4 years old) were analyzed. Thirty-three eyes (40.7%) suffered from central RVO (CRVO) and 48 (59.3%) from branch RVO (BRVO) with a median disease duration of 25 months (11–53 months). NPAs were found in 69.1% of eyes (*n* = 56, CRVO = 69.7%, *n* = 23; BRVO = 68.8%, *n* = 33) with a median size of 45.34 mm^2^ (11.39–81.39). Significant correlations between NPA size and CVs (*P* = 0.002, *r*_s_ = 0.344, confidence interval [CI] = 0.136–0.523), NPA size and GVs (*P* < 0.001, *r*_s_ = 0.49, CI = 0.302–0.64) as well as FAZ size and BCVA (*P* < 0.001, *r*_s_ = −0.429, CI = −0.593 to 0.231) were found.

**Conclusions:**

GVs may be indicators of underlying NPAs in RVO. For BRVO, CVs, IRF, IRH, and CS may be indicators for underlying NPAs. The 65 degrees WF-OCTA-imaging allows for precise noninvasive detection of vascular alterations in eyes with RVO and may serve as a useful adjunct in clinical practice.

Retinal vein occlusion (RVO) is the second most common retinal vascular disease after diabetic retinopathy.^1^ Generally, RVO can be divided into central RVO (CRVO), which in most literature includes hemi-CRVO (HCRVO), and branch RVO (BRVO). Overall, BRVO is the more common pathology with a worldwide prevalence of 0.4%, whereas CRVO affects approximately 0.08%.^2^ Prevalence of RVO in general is considered to increase with advanced age, although also the young population can be affected.^2^^,^^3^ Numerous retinal pathologies following RVO have been widely described, but are still not entirely understood. Retinal ischemia as a functionally relevant consequence of RVO affects roughly 20% of eyes. It is often associated with worse prognosis compared to non-ischemic RVO, both for central and peripheral visual acuity.^4^^,^^5^ Until now, the classification into ischemic and non-ischemic RVO has been performed via fluorescence angiography (FA). On FA, retinal non-perfusion the size of 10 disc areas is determined as an ischemic occlusion for CRVO; for BRVO it is 5 disc areas.^6^ With the introduction of optical coherence tomography angiography (OCTA) into clinical practice, a noninvasive, high resolution imaging modality has been made widely available, offering not only an alternative for the evaluation of retinal ischemia, but also valuable insights into other retinal vascular alterations.^7^^,^^8^ In many cases, it allows for better allocation of findings in and around the retina due to the addition of B-scans. However, so far, no gold standard has been established for OCTA image analysis. The most recent consensus on OCTA reporting guidelines defines ischemic CRVO as non-perfusion areas (NPAs) covering at least 30% of the acquired image, whereas no consensus could be reached for BRVO.^9^ However, smaller ischemic lesions might often be present even though the eyes were not characterized as ischemic. This study aims to analyze retinal vascular alterations and their association with NPAs following RVO using a single-capture wide-field (WF)-OCTA prototype and ultra-widefield color fundus (UWF-CF) imaging.

## Methods

Test subjects were recruited from the macular outpatient clinic at the Medical University of Vienna, Austria. Informed consent was obtained from every patient before inclusion. This prospective, cross-sectional study was performed in accordance with the International Conference on Harmonization of Technical Requirements for Registration of Pharmaceuticals for Human Use – Good Clinical Practice (ICH-GCP) guidelines.

All patients received routine clinical examination including visual acuity (Snellen), slit-lamp examination, OCTA (12 × 12 mm, PlexElite 9000; Carl Zeiss Meditec), WF-OCTA, and UWF-CF imaging (Clarus 700; Carl Zeiss Meditec). Patients with a confirmed diagnosis of BRVO, CRVO, or HCRVO and a minimum duration since disease onset of at least 12 weeks were included. The 12-week threshold was chosen to allow for large areas of pre- and intraretinal hemorrhage as well as large areas of macular edema to resolve for more accurate image grading. Images with severely reduced quality due to media opacity or motion artifacts due to poor compliance were discarded and those patients were excluded.

### Image Acquisition

WF-OCTA images were acquired using a custom built widefield swept source OCTA device developed at the Center for Medical Physics and Biomedical Engineering at the Medical University of Vienna. The device uses a 1.680 megahertz (MHz) laser, an A-scan rate of 2048 × 2 × 2048, a center wavelength of 1060 nm, an axial resolution of 9 µm, and a lateral resolution of 20 µm. Single capture images with a field of view (FOV) of 65 degrees or roughly 18 mm in diameter can be acquired in less than 15 seconds. Angiograms were visually enhanced using a deep learning-based algorithm for denoising.^10^^–^^12^ UWF-CF images were performed with Clarus 700 (Carl Zeiss Meditec) and OCTA images for size adjustment on the WF-OCTA prototype with PlexElite 9000 (Carl Zeiss Meditec).

### Image Preparation

A customized extended circular Early Treatment Diabetic Retinopathy Study (ETDRS)-grid was applied and centered on the fovea with the standard ETDRS diameter (1, 3, and 6 mm) and divided into superior, nasal, inferior, and temporal subfields. Two further rings were added with a diameter of 9 mm and 18 mm to mark the borders of the FOV of the WF-OCTA, thus resulting in an inner (1–3 mm) and outer ring (3–6 mm), with the addition of two extended rings (ER1 = 6–9 mm and ER2 = 9–18 mm).^12^ These extended rings were each split into eight quadrants (superior-nasal, nasal-superior, nasal-inferior, inferior-nasal, inferior-temporal, temporal-inferior, temporal-superior, and superior-temporal), allowing for a more detailed allocation of vascular alterations. To ensure each imaging modality is set at an equal scale and the central mm is applied over the fovea, affine transformation was performed on PlexElite, WF-OCTA, and CF images via Fiji (Fiji Is Just ImageJ), version 2.16.0, using the BigWarp plugin (version 9.1.0).^13^ The pixel size to the overlapped image was then adjusted to the scale approximation (12 × 12 mm) of the PlexElite image, allowing for an automatic transformation from pixels into mm^2^. Thus, all measurements on all imaging modalities were done in mm^2^ rather than pixels. Areas overshadowed by preretinal hemorrhage, hard exudates, or poor quality due to media opacity and motion artifacts were excluded from image grading as well as NPA measurement and subsequently subtracted from the total gradable area.

### Image Grading

**Figure 1.**
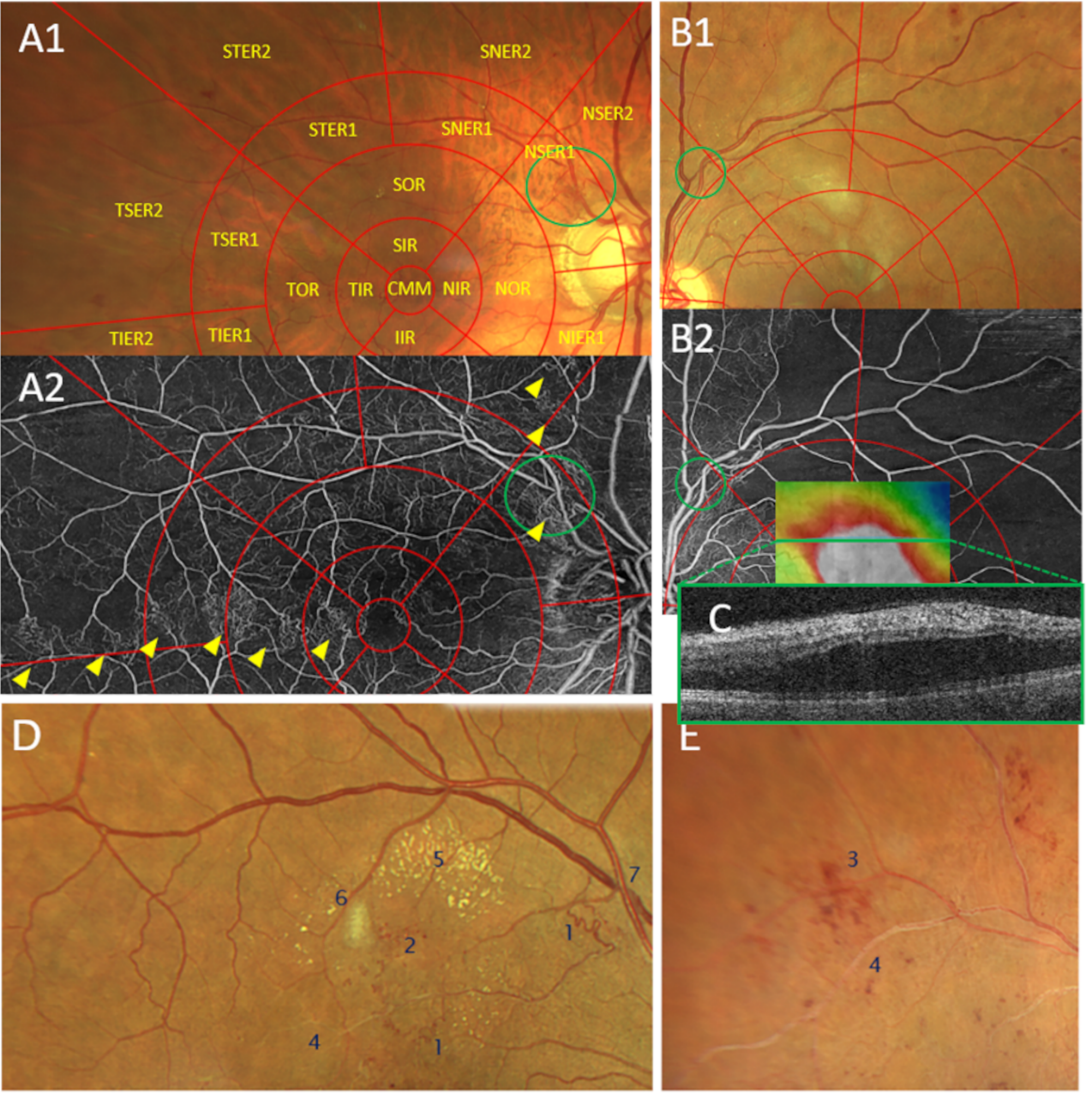
Parameters evaluated for image analysis. Images (**A–C**) show the assessment for location of occlusion, parameters on images (**D**) and (**E**) were graded manually. (**A1, A2, B1, B2**) Location of occlusion is marked within the *green circle*. Eyes were subsequently evaluated for collateral vessels (**A2**, *yellow arrows*), adjacent cotton wool spots, intraretinal hemorrhage (**B1**), nonperfusion areas (**B2**) or adjacent macular edema (thickness map on B2 overlapped with a Zeiss Cirrus 6000 en face image at initial presentation to better showcase extent of edema following the location of occlusion). In the case of **B**, no intraretinal fluid was present at the time of study enrollment due to anti-VEGF treatment. **C** represents a B-scan of a Zeiss Cirrus 6000 device at initial presentation, when macular edema was clearly visible. (**D,** **E**) Parameters evaluated on UWF-CF images: (1) collateral vessels, (2) microaneurysms, (3) intraretinal hemorrhage, (4) ghost vessels, (5) hard exudates, (6) cotton-wool spots, and (7) Crossing signs. Subfield labelling for **A1:** (clockwise from center to periphery): CMM, central millimeter; IIR, inferior IR; IOR, inferior OR; NIER1, nasal-inferior ER1; NIR, nasal IR; NOR, nasal OR; NSER1, nasal-superior ER1; NSER2, nasal-superior ER2; SIR, superior inner ring (IR); SOR, superior outer ring (OR); SNER1, superior-nasal extended ring 1 (ER1); SNER2, superior-nasal extended ring 2 (ER2); STER1, superior-temporal ER1; STER2, superior-temporal ER2; TIR, temporal inner ring; TIER1, temporal-inferior ER1; TIER2, temporal-inferior ER2; TOR, temporal OR; TSER1, temporal-superior ER1; TSER2, temporal-superior ER2.

**Figure 2.**
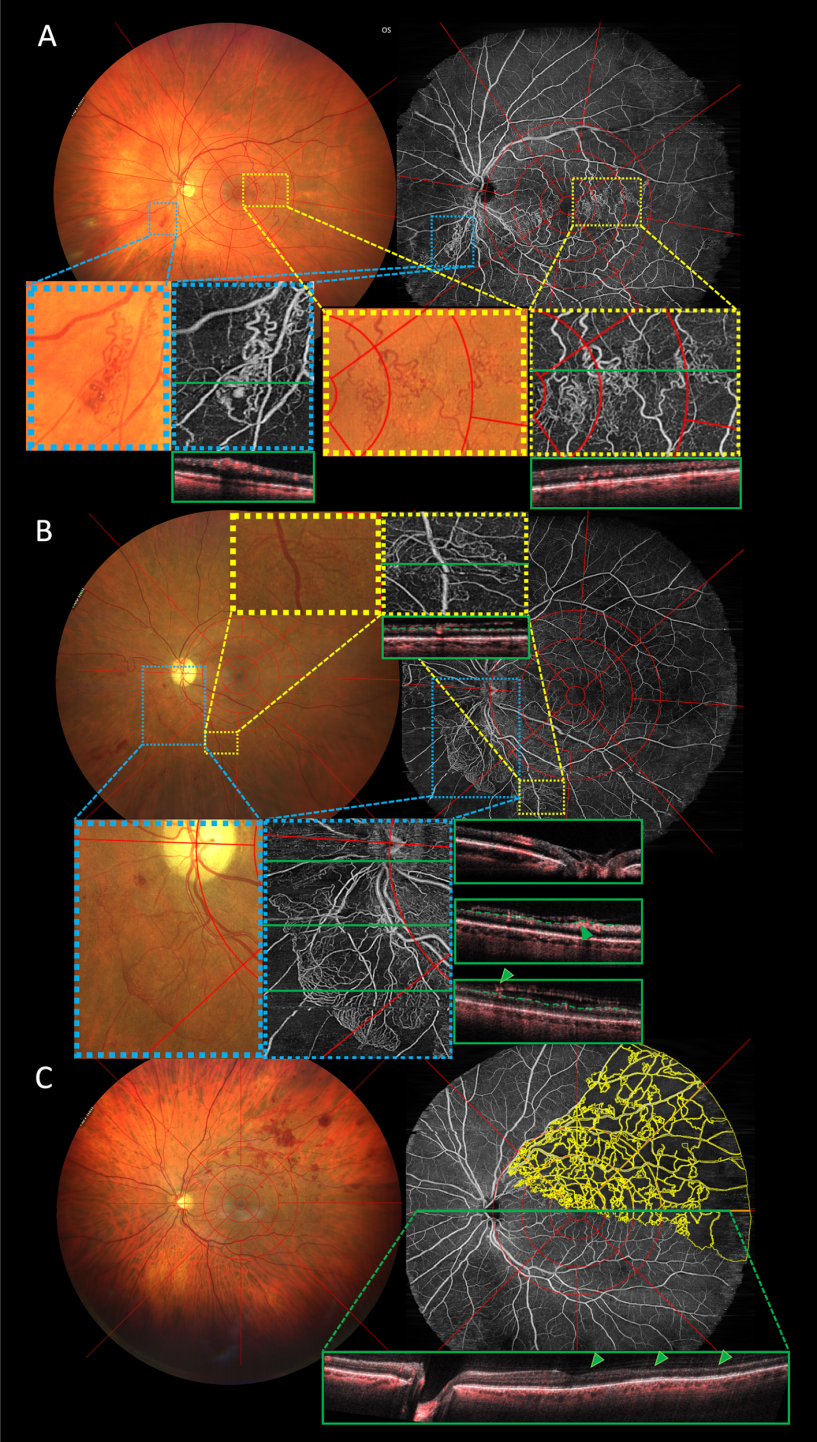
Side-to-side comparison of three eyes with retinal vein occlusion and retinal vascular alterations. Color fundus (CF) images are shown on the *left*, with corresponding widefield swept-source optical coherence tomography angiography (WF-OCTA) images on the *right*. (**A**) Patient with branch RVO (BRVO) on the lower vessel arch and collateral formation *highlighted with blue* and *yellow pop-out images*. Corresponding B-scans are marked as *green lines* on the en face image and depicted in the adjacent *green rectangle*. Because none of the vessels breach the internal limiting membrane (ILM) in the B-scans, vessel formations were classified as collateral vessels on WF-OCTA. (**B**) Patients with central RVO and both neovascularization of the disc (*highlighted in blue*) and elsewhere (*highlighted in yellow*). Both were verified on the WF-OCTA B-scans, where a clear breach of the ILM (*dashed green line*, *arrows* indicating sight of ILM breach) is visible in all corresponding B-scans. (**C**) Patient with BRVO in the upper vessel arch and expansive non-perfusion areas (NPAs) marked in *yellow* on the WF-OCTA via Fiji. *Arrows* on the B-scan (in *green*) point toward neuroretinal atrophy where NPAs are present.

Parameters graded on CF images include intraretinal hemorrhage (IRH), preretinal hemorrhage, spherical red dots corresponding to microaneurysms (MAs), collateral vessels (CVs; including CVs of the optic nerve head), cotton-wool spots (CW), neovascularization of the disc (NVD) or elsewhere (NVE), crossing signs (CS), presence of ghost-vessels (GVs; non-perfused retinal vessels remaining structurally visible on CF-images but without any flow signal on WF-OCTA images) and presence of hard exudates.^14^ WF-OCTA-grading consisted of CVs (dilated tortuous vessels within the retinal layers without breaching the internal limiting membrane), presence of NVD/NVE (vessel formation breaching the internal limiting membrane into the vitreous space), area of the foveal avascular zone (FAZ), presence of intraretinal (IRF) and subretinal fluid, and NPAs, which were labeled as such if their area exceeded 0.01 mm^2^. Skeletonized vessel density (SVD) was computed in Fiji by converting the WF-OCTA image to 8 bits, then binarizing it via Otsu auto thresholding, followed by skeletonizing the image. A ring extending approximately 0.5 mm beyond the optic disc was manually measured and excluded from analysis. SVD was defined as the total length of skeletonized vessels within a given area of measurement. To obtain this, each binarized vessel was reduced to a one-pixel-wide line. Whereas vessel density reflects the overall vasculature by incorporating both vessel size and length, SVD focuses solely on vessel length. This reduces the influence of large-caliber vessels and may therefore provide greater sensitivity to subtle changes in the retinal microvasculature.^15^^,^^16^ Location of occlusion was determined by evaluating both CF-images for CS and adjacent IRH, CWs, or GVs as well as WF-OCTA for NPAs, IRF (if not present at the moment of acquisition and the location of occlusion was not clear, then Zeiss Cirrus 6000 [Carl Zeiss Meditec] OCT images at the initial presentation at the macula outpatients’ clinic were taken into account; Fig. 1C), CVs, or neuroretinal atrophy corresponding to the location in question. A visualization of these parameters is shown in Figure 1, NPA grading is visualized in Figure 2. All of the above-mentioned parameters were first evaluated on the en face image and, if applicable, validated on the corresponding B-scans. As described by Stino et al. (2023), B-scans of areas on the en face images with no visible vascular flow signal were scanned for choroidal flow signal to ensure sufficient signal strength.^12^ All images were analyzed and graded by two experienced readers (authors J.K. and J.I.) and discrepancies were discussed with a retinal expert (author S.S.) until final consensus was reached.

### Statistical Analysis

Statistical analysis was performed separately for BRVO and CRVO (including HCRVO). Normally distributed values are expressed as mean ± standard deviation, otherwise as median (first quantile [Q1]; third quantile [Q3]). For both CRVO and BRVO, image findings were evaluated and compared between eyes with NPAs and without NPAs. Additionally, Spearman correlations and mixed models were applied to evaluate the relation among NPAs and demographic, functional, and descriptive parameters. The estimated correlation coefficient with 95% confidence intervals and *P* values were reported. Group comparisons were done using Mann-Whitney *U* tests and χ^2^ tests. Presence of vascular retinal changes among each subfield is schematically shown in topographic heatmaps. Due to the exploratory nature of this analysis, no multiplicity correction was performed. All statistical analysis was performed using R software version 4.0.3 (R Foundation for statistical computing, Vienna, Austria). The level of significance was set at α = 0.05.

## Results

A total of 116 patients with RVO were imaged using CF-imaging, WF-OCTA, and PlexElite OCTA. Thirty-five patients (30.2%) were excluded from this analysis. Reason for exclusion was disease duration <12 weeks (65.7%, *n* = 23), data processing issues resulting in faulty WF-OCTA images (14.3%, *n* = 5), insufficient image quality (11.4%, *n* = 4), or a disease diagnosis other than RVO (8.6%, *n* = 3). Thus, 81 eyes (48 right eyes and 33 left eyes) of 81 patients (women = 50.6%) with a mean age of 65 ± 12.4 years were included in this analysis. Of the eyes included, nine CF-images and four WF-OCTA images were affected by subfields deemed ungradable (number of subfields ungradable: 74/2025, 3.65% and 15/2025, 0.74%, respectively).

**Figure 3.**
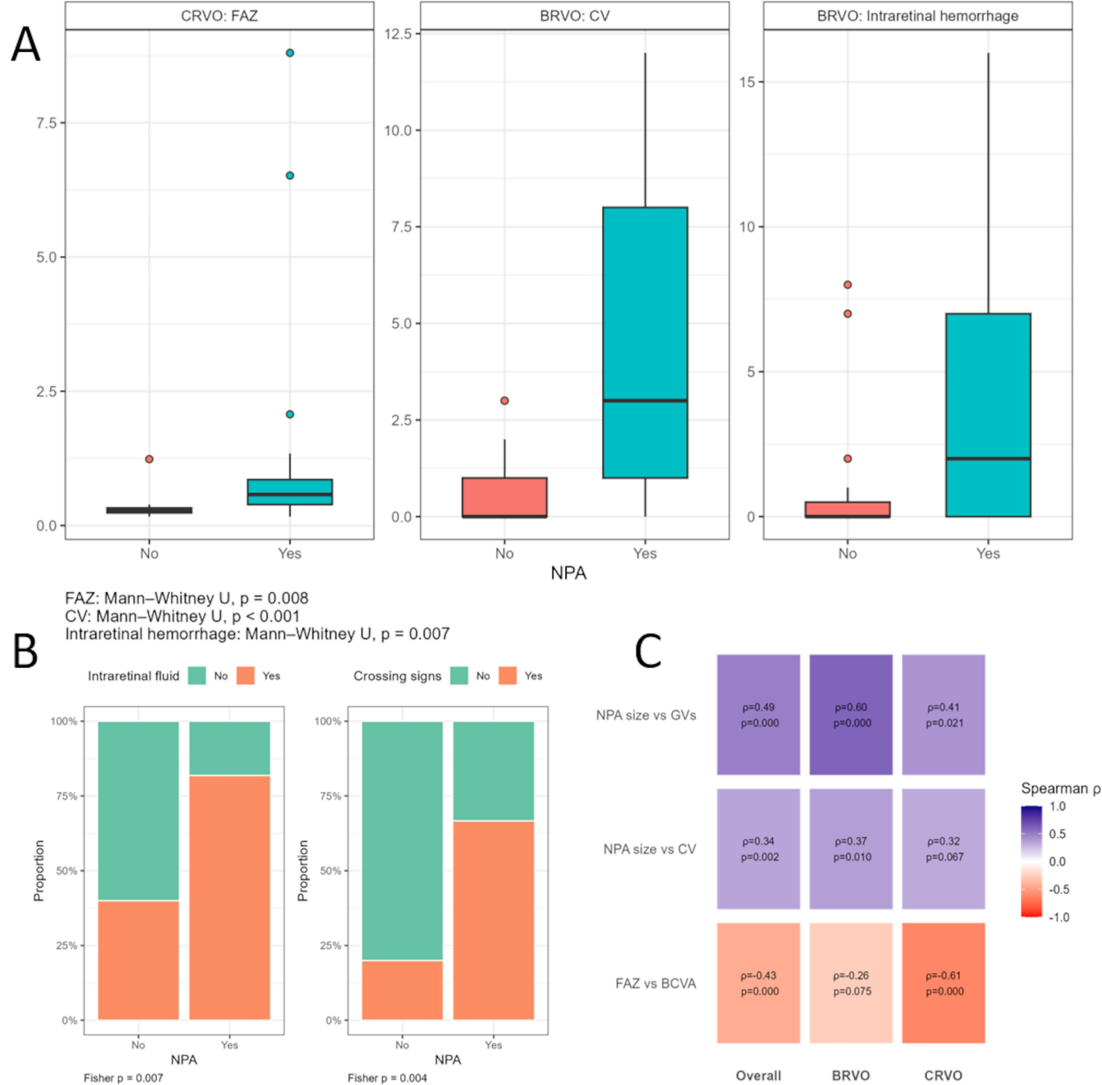
(**A**) Boxplots showing parameters with a significant difference between eyes with and without non-perfusion areas (NPA). (**B**) Barplots showing the significant differences in intraretinal fluid and crossing signs for eyes with branch retinal vein occlusion (BRVO) with and without NPAs. (**C**) Spearman correlation plots depicting correlations between NPA size and ghost vessels (GVs) and collateral vessels (CVs) as well as foveal avascular zone (FAZ) size and best corrected visual acuity (BCVA) for the overall study population, eyes with BRVO and central retinal vein occlusion (CRVO).

Forty-eight eyes (59.3%) suffered from BRVO and 33 (40.7%) from CRVO. Median disease duration for both groups was 25 months (11–53 months; BRVO = 23.7 [8.1–53.47] and CRVO = 29 [12–47]). Median number of anti-VEGF injections over the disease duration was 10 (3–18), with no significant difference between the groups (BRVO = 7.5 [2.75–17.25] and CRVO = 12 [6–18], *P* = 0.27). Of the 81 eyes included in the study, 88.9% (*n* = 72, BRVO = 85.4%, *n* = 41 and CRVO = 93.9%, *n* = 31) were pre-treated with anti-VEGF, whereas 5 eyes (BRVO = 4 and CRVO = 1) received treatment with extrafoveal sectorial laser photocoagulation targeting extensive NPAs. All eyes pretreated with laser coagulation were affected by extensive NPAs, whereas none were affected by NVD/NVE. Median best corrected visual acuity (BCVA; in Snellen) was 0.63 (0.32–0.8) for both groups (BRVO = 0.63 [0.48–0.8] and CRVO = 0.5 [0.2–0.63]). Mean central retinal thickness was 284.5 ± 186.2 µm (BRVO = 282.3 ± 194.1 µm and CRVO = 287.6 ± 176.9 µm). At the time of image acquisition, 48.1% (*n* = 39; BRVO = 43.8%, *n* = 21 and CRVO = 54.5%, *n* = 18) of patients suffered from hypertension, 13.6% (*n* = 11; BRVO = 12.5%, *n* = 6 and CRVO = 15.2%, *n* = 5) from diabetes mellitus and 12.3% (*n* = 10; BRVO = 6.3%, *n* = 3 and CRVO = 21.2%, *n* = 7) from cardiovascular disease. Details for each group are shown in Table 1, significant differences are shown in Figures 3A and 3B. Correlations evaluating imaging parameters and disease duration as well as anti-VEGF were significant for CW in eyes with BRVO (*P* < 0.001; Supplementary Table S1).

**Table 1.** Group Comparisons Between Eyes With and Without Nonperfusion Areas for Both Central- and Branch Retinal Vein Occlusion

	BRVO				CRVO			
	Cohort, *n* = 48	No NPA, *n* = 15	NPA, *n* = 33	*P* Value	Cohort, *n* = 33	No NPA, *n* = 10	NPA, *n* = 23	*P* Value
IVOM median (Q1; Q3)	7.5 (2.75; 17.25)	6 (3.5; 23.5)	8 (2; 13)	0.540^*^	12 (6; 18)	9 (5.25; 12)	14 (6; 19.5)	0.377^*^
BCVA study eye median (Q1; Q3)	0.63 (0.48; 0.8)	0.8 (0.45; 0.9)	0.63 (0.5; 0.8)	0.450^*^	0.5 (0.2; 0.63)	0.63 (0.34; 0.76)	0.4 (0.15; 0.63)	0.365^*^
CRT median (Q1; Q3)	223.5 (205.5; 268.8)	223 (214.5; 263)	224 (201; 267)	0.815^*^	222 (193; 267)	230 (216.75; 272)	208 (181.5; 263.5)	0.182^*^
Intraretinal fluid n	33	6	27	0.007^***^	19	5	14	0.707^***^
Subretinal fluid n	6	0	6	^**^	3	1	2	^**^
CV median (Q1; Q3)	1.5 (0; 6)	0 (0; 1)	3 (1; 8)	<0.001^*^	0 (0; 4)	0 (0; 0.75)	1 (0; 4)	0.113^*^
FAZ median (Q1; Q3)	0.37 (0.3; 0.45)	0.36 (0.3; 0.41)	0.39 (0.32; 0.57)	0.336^*^	0.44 (0.28; 0.81)	0.28 (0.24; 0.33)	0.58 (0.39; 0.86)	0.008^*^
Microaneurysms median (Q1; Q3)	3 (0; 8)	2 (0; 4)	5 (0; 9)	0.225^*^	6 (1; 11)	5 (0.75; 9.5)	7 (1.5; 11.5)	0.478^*^
Intraret. Hemorrhage median (Q1; Q3)	1 (0; 4.5)	0 (0; 0.5)	2 (0; 7)	0.007^*^	2 (1; 8)	2 (0.25; 6.75)	3 (1.5; 8)	0.464^*^
Hard exudates median (Q1; Q3)	4 (0; 9)	2 (0; 4)	4 (0; 9)	0.410^*^	2 (0; 10)	1.5 (0; 6.5)	3 (0; 10)	0.703^*^
CW-spots median (Q1; Q3)	0 (0; 1)	0 (0; 1)	0 (0; 1)	0.873^*^	0 (0; 0)	0 (0; 0)	0 (0; 0)	0.884^*^
CV CF median (Q1; Q3)	0 (0; 2)	0 (0; 0)	0 (0; 3)	0.052^*^	0 (0; 1)	0.5 (0; 1)	0 (0; 1.5)	0.865^*^
Crossing signs *n*	25	3	22	0.004^***^	8	3	5	0.673^***^
Ghost vessels *n*	17	0	17	^**^	6	0	6	^**^

For the group comparison of continuous variables, the *P* values from the Mann-Whitney *U* test are reported, whereas for categorical variables, the *P* value from the Chi-square or Fisher’s exact test is reported.

Statistical test performed: ^*^ Mann-Whitney *U* test, ^**^ chi squared test; ^***^ Fisher's exact test.

Categorial variables were summarized in contingency tables. If expected cell counts were sufficient, *P* values were computed from the chi-square test. If any expected count was <5 but all categories were represented, we used Fisher's exact test. In cases of complete separation (i.e. a category with zero observations) or very sparse data (e.g. group size of 1), we did not report a *P* value. The significance level is α = 0.05.

BCVA, best corrected visual acuity; CV, collateral vessel; CV CF, collateral vessel evaluated on color fundus; CW-spots, cotton-wool spots; CRT, central retinal thickness; FAZ, foveal avascular zone (in mm^2^); IVOM, intravitreal ocular medication.

The significance of the figures in bold represent *P* < 0.05.

### Image Findings BRVO

**Figure 4.**
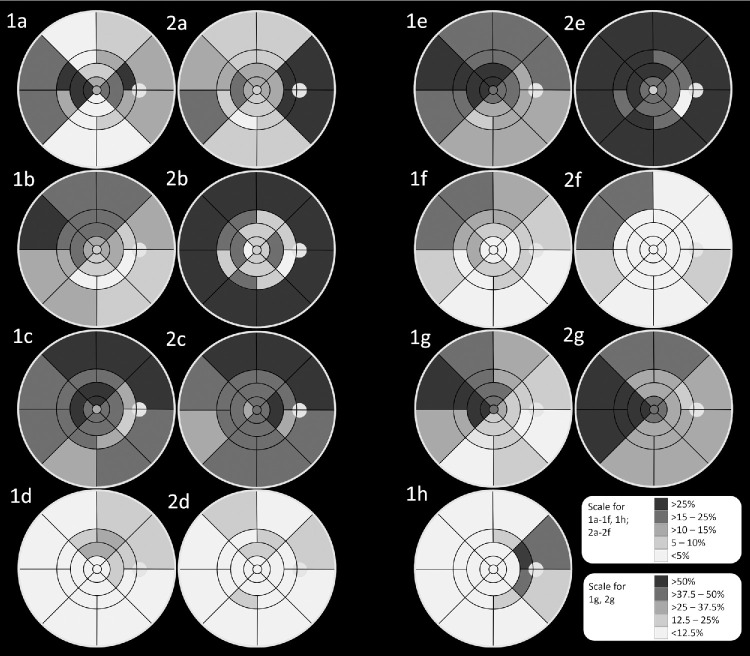
Visualization of image parameters found on widefield optical coherence tomography angiography and ultra-widefield color fundus imaging for branch retinal vein occlusion (**1a–h**) and central retinal vein occlusion (**2a–g**). All images are visualized as right eyes. (**1a, 2a**) Subfields affected by collateral vessels; (**1b, 2b**) subfields affected by intraretinal hemorrhage; (**1c, 2c**) subfields affected by hard exudates; (**1d, 2d**) subfields affected by cotton wool spots; (**1e, 2e**) subfields affected by microaneurysms; (**1f, 2f**) subfields affected by ghost vessels; (**1g, 2g**) subfields affected by non-perfusion areas; and (**1h**) location of retinal occlusion.

For detailed image findings on WF-OCTA and UWF-CF refer to Figure 4 and Table 2. Of the 48 eyes, location of occlusion was found exclusively in the ER1 (62.5%, *n* = 30) and ER2 (33.3%, *n* = 16). The most affected subfield was the inner nasal-superior (27.1%, *n* = 13), followed by the outer nasal-superior (22.9%, *n* = 11) and inner nasal inferior (18.8%, *n* = 9). For 2 eyes (4.2%), the exact location of occlusion was not assignable to a single subfield. Overall, 60.4% (*n* = 29) of BRVOs were located in the upper and 35.4% (*n* = 17) in the lower hemisphere.

**Table 2.** Image Parameters Found on Widefield Optical Coherence Tomography Angiography and Ultra-Widefield Color Fundus Imaging for Branch Retinal Vein Occlusion (BRVO) and Central Retinal Vein Occlusion (CRVO)

	Total	CV *N* (%) Present	MA *N* (%) Present	HE *N* (%) Present	CW *N* (%) Present	IRH *N* (%) Present	GV *N* (%) Present	NPA (mm^2^) *N*; Median (Q1; Q3)
BRVO, *N* = 48								
CMM		6 (12.5)	8 (16.67)	6 (12.5)	0	3 (6.25)	0	21; 0.14 (0.07; 0–27)
IR	*N* (%) present	16 (33.33)	18 (37.5)	17 (35.42)	2 (4.17)	10 (20.83)	3 (6.25)	26 (54.17)
	Median (Q1; Q3) present	2 (1; 3)	2 (1; 3)	3 (1; 4)	2 (1; 2)	2 (2; 2)	1 (1; 1)	1.27 (0.7; 1.92)
	Superior	7 (14.58)	9 (18.75)	14 (29.17)	2 (4.17)	6 (12.5)	1 (2.08)	22; 0.46 (0.1; 0.87)
	Nasal	10 (20.83)	10 (20.83)	11 (22.92)	0	5 (10.42)	0	16; 0.17 (0.06; 0.32)
	Inferior	2 (4.17)	8 (16.67)	9 (18.75)	0	3 (6.25)	2 (4.17)	10; 0.63 (0.19; 1.08)
	Temporal	14 (29.17)	12 (25)	10 (20.83)	1 (2.08)	7 (14.58)	0	26; 0.33 (0.18; 0.57)
OR	*N* (%) present	18 (37.5)	25 (52.08)	21 (43.75)	9 (18.75)	14 (29.17)	10 (20.83)	31 (64.58)
	Median (Q1; Q3) present	2 (1;2)	2 (1; 2)	2 (1; 3)	1 (1; 1)	2 (1; 2)	1 (1; 2)	2.82 (1.28; 5.54)
	Superior	4 (8.33)	9 (18.75)	13 (27.08)	5 (10.42)	9 (18.75)	4 (8.33)	22; 1.71 (0.4; 2.57)
	Nasal	8 (16.67)	10 (20.83)	11 (22.92)	3 (6.25)	6 (12.5)	1 (2.08)	12; 0.46 (0.32; 0.88)
	Inferior	1 (2.08)	7 (14.58)	11 (22.92)	2 (4.17)	3 (6.25)	3 (6.25)	8; 0.77 (0.4; 3.69)
	Temporal	16 (33.33)	18 (37.5)	14 (29.17)	1 (2.08)	9 (18.75)	5 (10.42)	28; 1.12 (0.55; 1.76)
ER1	*N* (%) present	27 (56.25)	25 (52.08)	17 (35.42)	6 (12.5)	15 (31.25)	14 (29.17)	30 (62.5)
	Median (Q1; Q3) present	2 (1; 2)	2 (1; 3)	4 (2; 5)	2 (1; 2)	2 (1; 4)	2 (2; 3)	6.78 (2.4; 11.24)
	Superior-temporal	2 (4.17)	8 (16.67)	10 (20.83)	1 (2.08)	9 (18.75)	7 (14.58)	19; 3.17 (1.59; 4.09)
	Superior-nasal	7 (14.58)	8 (16.67)	8 (16.67)	5 (10.42)	9 (18.75)	7 (14.58)	15; 3.24 (1.02; 3.74)
	Nasal-superior	13 (27.08)	6 (12.5)	6 (12.5)	3 (6.25)	4 (8.33)	4 (8.33)	11; 0.57 (0.29; 0.91)
	Nasal-Inferior	5 (10.42)	5 (10.42)	4 (8.33)	0	1 (2.08)	3 (6.25)	4; 0.05 (0.02; 0.18)
	Inferior-nasal	3 (6.25)	5 (10.42)	7 (14.58)	0	1 (2.08)	4 (8.33)	6; 1 (0.46; 2.3)
	Inferior-temporal	1 (2.08)	3 (6.25)	9 (18.75)	1 (2.08)	2 (4.17)	2 (4.17)	5; 3.44 (3.19; 4)
	Temporal-inferior	7 (14.58)	7 (14.58)	9 (18.75)	0	5 (10.42)	2 (4.17)	13; 0.89 (0.51; 2.98)
	Temporal-superior	14 (29.17)	15 (31.25)	10 (20.83)	0	8 (16.67)	5 (10.42)	27; 2.05 (0.86; 3.43)
ER2	N (%) present	20 (41.67)	23 (47.92)	30 (62.5)	8 (16.67)	26 (54.17)	15 (31.25)	29 (60.42)
	Median (Q1; Q3) present	1 (1; 2)	4 (1; 4)	3 (1; 4)	1 (1; 2)	3 (2; 4)	2 (2; 2)	41.37 (13.64; 57.7)
	Superior-temporal	1 (2.08)	11 (22.92)	15 (31.25)	2 (4.17)	12 (25)	10 (20.83)	20; 18.4 (3.19; 23.7)
	Superior-nasal	3 (6.25)	11 (22.92)	17 (35.42)	4 (8.33)	9 (18.75)	5 (10.42)	16; 13.87 (3.03; 19.73)
	Nasal-superior	6 (12.5)	10 (20.83)	17 (35.42)	3 (6.25)	6 (12.5)	3 (6.25)	10; 0.31 (0.2; 2.71)
	Nasal-inferior	5 (10.42)	7 (14.58)	9 (18.75)	2 (4.17)	4 (8.33)	2 (4.17)	4; 1.26 (0.93; 3.24)
	Inferior-nasal	1 (2.08)	7 (14.58)	10 (20.83)	0	4 (8.33)	0	6; 3.58 (1.66; 5.15)
	Inferior-temporal	0	5 (10.42)	7 (14.58)	0	6 (12.5)	1 (2.08)	4; 15.99 (11.42; 17.64)
	Temporal-inferior	9 (18.75)	10 (20.83)	8 (16.67)	0	6 (12.5)	4 (8.33)	17; 3.76 (2.15; 10.91)
	Temporal-superior	10 (20.83)	15 (31.25)	11 (22.92)	1 (2.08)	16 (33.33)	8 (16.67)	25; 17.02 (10.03; 19.91)
Total	*N* (%) present	31 (64.58)	32 (66.67)	34 (70.83)	16 (33.33)	27 (56.25)	17 (35.42)	33 (68.75)
	Median (Q1; Q3) present	4 (2; 8)	6.5 (3; 9)	6.5 (3.25; 9.75)	1.5 (1; 5.75)	3 (2; 8.5)	5 (3; 6)	42.59 (10.01; 71.76)
CRVO, *N* = 33								
CMM		2 (6.06)	1 (3.03)	6 (18.18)	0	2 (6.06)	0	14; 0.28 (0.15; 0.36)
IR	*N* (%) present	5 (15.15)	11 (33.33)	9 (27.27)	1 (3.03)	4 (12.12)	0	18 (54.54)
	Median (Q1; Q3) present	3 (2; 4)	1 (1; 3)	2 (1; 3)	10 (10;10)	1.5 (1; 2)		1.03 (0.39; 3.24)
	Superior	4 (12.12)	4 (12.12)	6 (18.18)	1 (3.03)	2 (6.06)	0	14; 0.32 (0.11; 1.14)
	Nasal	4 (12.12)	4 (12.12)	5 (15.15)	1 (3.03)	2 (6.06)	0	14; 0.3 (0.18; 1.05)
	Inferior	2 (6.06)	6 (18.18)	5 (15.15)	1 (3.03)	2 (6.06)	0	14; 0.46 (0.19; 0.98)
	Temporal	4 (12.12)	7 (21.21)	4 (12.12)	1 (3.03)	1 (3.03)	0	18; 0.27 (0.15; 1.04)
OR	*N* (%) present	6 (18.18)	14 (42.42)	13 (39.39)	2 (6.06)	8 (24.24)	0	20 (60.6)
	Median (Q1; Q3) present	2 (1; 4)	2 (1; 3)	2 (2; 3)	2 (2; 2)	2 (1; 2)		1.78 (0.73; 9.2)
	Superior	4 (12.12)	10 (30.3)	6 (18.18)	2 (6.06)	3 (9.09)	0	12; 1.39 (0.44; 4.35)
	Nasal	4 (12.12)	6 (18.18)	9 (27.27)	1 (3.03)	6 (18.18)	0	10; 1.39 (0.32; 3.61)
	Inferior	2 (6.06)	6 (18.18)	7 (21.21)	1 (3.03)	2 (6.06)	0	12; 2.18 (0.83; 4.2)
	Temporal	5 (15.15)	10 (30.3)	8 (24.24)	0	5 (15.15)	0	18; 1.32 (0.44; 4.19)
ER1	*N* (%) present	12 (36.36)	19 (57.58)	14 (42.42)	3 (9.09)	10 (30.30)	3 (9.09)	19 (57.58)
	Median (Q1; Q3) present	2 (1; 3)	3 (2; 4)	4 (2; 5)	2 (2.3)	2 (1; 4)	1 (1; 1)	4.48 (2.25; 17.58)
	Superior-temporal	2 (6.06)	11 (33.33)	8 (24.24)	1 (3.03)	5 (15.15)	0	12; 2.65 (1.68; 3.22)
	Superior-nasal	2 (6.06)	6 (18.18)	6 (18.18)	2 (6.06)	2 (6.06)	0	12; 1.06 (0.66; 2.6)
	Nasal-superior	9 (27.27)	7 (21.21)	5 (15.15)	1 (3.03)	2 (6.06)	1 (3.03)	8; 1.46 (0.69; 1.13)
	Nasal-inferior	9 (27.27)	0	4 (12.12)	0	1 (3.03)	0	9; 0.61 (0.49; 2.46)
	Inferior-nasal	3 (9.09)	6 (18.18)	6 (18.18)	1 (3.03)	3 (9.09)	0	11; 1.04 (0.53; 2.55)
	Inferior-temporal	1 (3.03)	8 (24.24)	5 (15.15)	2 (6.06)	6 (18.18)	1 (3.03)	9; 3.05 (2.83; 3.6)
	Temporal-inferior	3 (9.09)	7 (21.21)	8 (24.24)	0	3 (9.09)	0	17; 1.72 (0.52; 3.97)
	Temporal-superior	4 (12.12)	12 (36.36)	8 (24.24)	0	7 (21.21)	1 (3.03)	18; 2.12 (0.47; 3.95)
ER2	*N* (%) present	15 (45.45)	25 (75.76)	18 (54.55)	4 (12.12)	19 (57.58)	6 (18.18)	20 (60.61)
	Median (Q1; Q3) present	2 (2; 2)	6 (3; 7)	3 (2; 6)	1 (1; 2)	4 (2; 8)	2 (2; 4)	56.35 (23.77; 106.37)
	Superior-temporal	3 (9.09)	21 (63.64)	13 (39.39)	3 (9.09)	13 (39.39)	5 (15.15)	16; 16.13 (6.17; 24.71)
	Superior-nasal	3 (9.09)	16 (48.48)	12 (36.36)	1 (3.03)	14 (42.42)	0	14; 11.02 (9; 21.78)
	Nasal-superior	11 (33.33)	17 (51.52)	13 (39.39)	2 (6.06)	11 (33.33)	0	10; 13.21 (6.36; 22.92)
	Nasal-inferior	9 (27.27)	13 (39.39)	6 (18.18)	1 (3.03)	11 (33.33)	2 (6.06)	10; 12.36 (9.48; 19.75)
	Inferior-nasal	2 (6.06)	12 (36.36)	8 (24.24)	1 (3.03)	11 (33.33)	1 (3.03)	10; 14.57 (3.79; 19.4)
	Inferior-temporal	2 (6.06)	13 (39.39)	5 (15.15)	1 (3.03)	12 (36.36)	1 (3.03)	10; 11.52 (6.02; 15.55)
	Temporal-inferior	5 (15.15)	19 (57.58)	4 (12.12)	0	11 (33.33)	2 (6.06)	17; 15.89 (7.21; 20.77)
	Temporal-superior	4 (12.12)	18 (54.55)	8 (24.24)	0	17 (51.52)	5 (15.15)	18; 19.45 (11.32; 21.6)
Total	*N* (%) present	16 (48.48)	26 (78.79)	21 (63.64)	6 (18.8)	26 (78.79)	6 (18.18)	23 (69.7)
	Median (Q1; Q3) present	4 (2; 9)	9 (5; 12)	9 (3; 11)	1.5 (1; 6)	4 (2; 8)	3 (2.25; 3.75)	48.09 (12.71; 128.02)

For values with values present/not present, absolute and relative frequencies are given. For variables where the number of subfields affected are counted as well as for variable NPA (mm^2^), median (Q1; Q3) is given for variables with values >0. Total is the sum of the corresponding field values (0 = not present /1 = present).

CMM, central millimeter; CV, collateral vessel; CW, cotton-wool spots; ER1, extended ring 1; ER2, extended ring 2; GV, ghost vessel; HE, hard exudates; IR, inner ring; IRH, intraretinal hemorrhage; MA, microaneurysms; NPA, nonperfusion area; OR, outer ring.

CVs were found in 64.6% (*n* = 31) of eyes. Where CVs were present, median total number of subfields affected by CVs was 4 (2; 8) and 2 (1; 3), 2 (1; 2), 2 (1; 2), and 1 (1; 2) in the inner ring (IR), outer ring (OR), ER1, and ER2, respectively.

Microaneurysms were found in 66.7% (*n* = 32) of eyes. Where MAs were present, the median total number of subfields affected by MAs was 6.5 (3; 9) and 2 (1; 3), 2 (1; 2), 2 (1; 3), and 4 (1; 4) in the IR, OR, ER1, and ER2, respectively.

Intraretinal hemorrhage was found in 56.3% (*n* = 27) of eyes. Where IRH was present, median total number of subfields affected by IRH was 3 (2; 8.5) and 2 (2; 2), 2 (1; 2), 2 (1; 4), and 3 (2; 4) in the IR, OR, ER1, and ER2, respectively.

GVs were found in 35.4% (*n* = 17) of eyes. Where GVs were present, median total number of subfields affected by GVs was 5 (3; 6) and 1 (1; 1), 1 (1; 2), 2 (2; 3) and 2 (2; 2) in the IR, OR, ER1, and ER2, respectively.

No NVs were found in eyes with BRVO.

### Image Findings CRVO

For detailed image findings on WF-OCTA and UWF-CF refer to Figure 4 and Table 2. CVs were found in 48.5% (*n* = 16) of eyes. Where CVs were present, median total number of subfields affected by CVs was 4 (2; 9) and 3 (2; 4), 2 (1; 4), 2 (1; 3) and 2 (2; 2) in the IR, OR, ER1, and ER2, respectively.

Microaneurysms were found in 78.8% (*n* = 26) of eyes. Where MAs were present, median total number of subfields affected by MAs was 9 (5; 12) and 1 (1; 3), 2 (1; 3), 3 (2; 4), and 6 (3; 7) in the IR, OR, ER1, and ER2, respectively.

Intraretinal hemorrhage was found in 78.8% (*n* = 26) of eyes. Where IRH was present, median total number of subfields affected by IRH was 4 (2; 8) and 1.5 (1; 2), 2 (1; 2), 2 (1; 4) and 4 (2; 8) in the IR, OR, ER1, and ER2, respectively.

GVs were found in 18.2% (*n* = 6) of eyes. Where GVs were present, median total number of subfields affected by GVs was 3 (2.25; 3.75) and 0 (0; 0), 0 (0; 0), 1 (1; 1) and 2 (2; 4) in the IR, OR, ER1, and ER2, respectively.

In total, NVs were detected in two eyes with CRVO – one eye with both NVD and NVE (in the inferior and nasal quadrant spanning from the papilla to the far periphery) and one with solely NVD (see Fig. 2).

### NPA Features and Measurements

In total, NPAs were found in 69.1% of eyes (*n* = 56; CRVO = 69.7%, *n* = 23 and BRVO = 68.8%, *n* = 33). Median NPA-size was 45.34 mm^2^ (11.39–81.39). Eyes with and without NPAs were compared for functional (BCVA) and clinical data. For BRVO, eyes with NPAs showed significantly more retinal changes than eyes without NPAs, such as the presence of IRF (*P* = 0.007), intraretinal hemorrhage (*P* = 0.007), more CVs (*P* < 0.001), and the detection of CS (*P* = 0.004). For CRVO, FAZ size was significantly larger in eyes with NPAs (*P* = 0.008). For both BRVO and CRVO, GVs were only found if NPAs were present. Details are shown in Table 1.

Overall, significant correlations between NPA size and CVs (*P* = 0.002, *r*_s_ = 0.344, CI = 0.136–0.523), NPA size and GVs (*P* < 0.001, *r*_s_ = 0.49, CI = 0.302–0.64) as well as FAZ size and BCVA (*P* < 0.001, *r*_s_ = −0.429, CI = −0.593 to 0.231) were found (Fig. 3C; Table 3). Significant association of GV with NPA did not change after adjusting for disease duration (Supplementary Table S2). Inclusion of confounders (hypertension, diabetes mellitus, and cardiovascular disease) did not change significance of the associations of CV and GV with NPA (*P* < 0.001, respectively). Association between BCVA and FAZ remained significant in the overall cohort (*P* < 0.001) and in CRVO (*P* = 0.004), but was not significant in BRVO (*P* = 0.117; see Table 3, Supplementary Table S3). A linear regression model analyzing the association of CVs with NPA showed that in the overall cohort, each additional CV was associated with a 35% increase in (1 + NPA, *P* = 0.002). Location (peripheral >6–18 mm versus posterior pole 0–6 mm) and the interaction between location and CV number were not significant (*P* = 0.768 and *P* = 0.851, respectively), indicating a similar relationship between collateral vessels and NPA across locations. CV on the optic disc showed no evidence of association with NPA (exp(β) = 1.02 per unit increase, 95% CI = 0.61–1.72, *P* = 0.94). Details can be found in Supplementary Table S4. Regarding SVD, both BRVO and CRVO eyes showed a similar pattern. For BRVO, SVD was highest in the nasal quadrant (11.97 ± 3.27), followed by the inferior (10.2 ± 3.89), superior (8.68 ± 3.12), and temporal quadrant (8.52 ± 2.73). For CRVO, again SVD was highest in the nasal quadrant (8.87 ± 3.1), followed by the inferior (7.35 ± 2.31), superior (6.92 ± 2.74), and temporal quadrants (6.67 ± 3.01).

**Table 3.** Summary of Correlations (Spearman and Pearson) and Linear Regressions (Unadjusted and Adjusted Coefficients With 95% CI and *P* Values) Shown for the Overall Cohort, Branch Retinal Vein Occlusion (BRVO), and Central Retinal Vein Occlusion (CRVO)

Outcome and Covariate	Cohort	Spearman rho (p)	Pearson *r* (*P*)	Unadjusted Coefficient (95% CI), *P*	Adjusted Coefficient (95% CI), *P*
Log (1+NPA size) ∼ GV	Overall	0.49 (***P* < 0.001**)	0.45 (***P* < 0.001**)	0.37 (0.21 to 0.54), ***P* < 0.001**	0.37 (0.2 to 0.55), ***P* < 0.001**
	BRVO	0.6 (***P* < 0.001**)	0.55 (***P* < 0.001**)	0.36 (0.2 to 0.52), ***P* < 0.001**	0.38 (0.21 to 0.56), ***P* < 0.001**
	CRVO	0.41 (***P* = 0.021**)	0.4 (***P* = 0.022**)	0.65 (0.1 to 1.2), ***P* = 0.022**	0.82 (0.11 to 1.52), ***P* = 0.025**
Log (1+NPA size) ∼ CV	Overall	0.34 (***P* = 0.002**)	0.36 (***P* < 0.001**)	0.15 (0.06 to 0.24), ***P* < 0.001**	0.16 (0.07 to 0.25), ***P* < 0.001**
	BRVO	0.37 (***P* = 0.01**)	0.35 (***P* = 0.015**)	0.17 (0.03 to 0.31), ***P* = 0.015**	0.18 (0.03 to 0.32), ***P* = 0.017**
	CRVO	0.32 (*P* = 0.067)	0.38 (***P* = 0.029**)	0.14 (0.01 to 0.26), ***P* = 0.029**	0.16 (0.03 to 0.29), ***P* = 0.017**
FAZ size ∼ BCVA	Overall	−0.43 (***P* < 0.001**)	−0.39 (***P* < 0.001**)	−1.42 (−2.17 to −0.66), ***P* < 0.001**	−1.39 (−2.15 to −0.64), ***P* < 0.001**
	BRVO	−0.26 (*P* = 0.075)	−0.32 (***P* = 0.028**)	−0.28 (−0.53 to −0.03), ***P* = 0.028**	−0.21 (−0.48 to 0.06), *P* = 0.117
	CRVO	−0.61 (***P* < 0.001**)	−0.46 (***P* = 0.007**)	−2.42 (−4.13 to −0.71), ***P* = 0.007**	−2.66 (−4.38 to −0.94), ***P* = 0.004**

BCVA, best corrected visual acuity; CV, collateral vessel; FAZ size, size of the foveal avascular zone; GV, ghost vessel; NPA size, size of nonperfusion areas.

For more detail see Supplementary Table S3.

The significance of the figures in bold represent *P* < 0.05.

## Discussion

In this cross-sectional, observational study, we evaluated retinal vascular alterations in eyes suffering from both BRVO and CRVO and compared our findings between eyes with and without the presence of NPAs. Additionally, we aimed to showcase correlations and a detailed description of the image findings shown on single capture WF-OCTA (see Fig. 4).

FA is still seen as the gold standard for evaluation of the retinal vessel structure. In this analysis, we used single capture-WF-OCTA, a fast, noninvasive way to gather information about the retinal microstructure even more precise than FA.^7^^,^^17^^,^^18^ In addition to NPA detection, it is an important tool to gather more information about the retinal vessel structure, such as detection of NVs and CVs, as the addition of a B-scan allows for more exact allocation of the object in question to a specific retinal layer.^19^^,^^20^

In our study, CVs seem to be the best predictor for the presence of retinal NPAs in eyes with BRVO, a finding contrary to earlier research evaluating CVs and NPAs.^20^ This may be due to our significantly larger field of view (18 × 18 mm) compared to the 12 × 12 mm used by Rudnick et al. When looking at the topographic distribution of CVs (see Fig. 4), a large extent is located in the ER2, especially in the temporal and nasal quadrants (median CVs in ER2: CRVO = 2 [2; 2], BRVO = 1 [1; 2]). Rudnick et al. identified CVs in 42.9% (12/28) of eyes with BRVO, 45.5% (25/55) located outside the macular region. We found CVs in 64.58% (*n* = 31) of eyes. Most studies evaluating CVs are done on a smaller FOV.^20^^,^^21^ Generally, it can be difficult to define where a CV-bundle ends and a new bundle begins, which is why we focused on describing subfields affected by CVs rather than actual bundles. Still, a median of 4 (2; 8) subfields affected by CVs were found in 31 eyes with BRVO, suggesting a higher number of CVs than Rudnick et al. found. Therefore, an increased FOV is important to properly analyze retinal alterations in both CRVO and BRVO. Location of CV (central versus peripheral) did not show to be a significant predictor of NPA size. However, for BRVO, CVs were found mostly in the nasal quadrant around the location of occlusion and in the temporal quadrant (see Fig. 4 1a), bridging the area of intact retinal vessels and NPA areas. Thus, location of NPA often extended far beyond the quadrants affected by CVs (see Fig. 4 1g). For CRVO, CVs were mostly found around the optic disc (see Fig. 4 2a) and were not significant in regard to NPA formation. Arrigo et al. (2021) have previously described CVs to be more spread out in eyes affected by BRVO than CRVO.^22^ In their study, of 18 eyes affected by CRVO and presence of peripheral capillary nonperfusion, only 38.8% (*n* = 7) were affected by CVs, also indicating that CVs in CRVO might not be indicative of underlying NPAs. They have also described a generalized lower vessel density on 9 × 9 mm OCTA images in eyes affected by CRVO compared to BRVO, a finding coherent with our SVD results.^22^ Siying et al. (2022) compared WF-OCTA images to WF-FA images of eyes with BRVO and found that WF-OCTA revealed NPAs more accurately than WF-FA and was better suited for CV detection.^23^ In our study, we found macular edema was significantly more prevalent in eyes with BRVO where NPAs were present. This was in part shown before, as a previous study using OCTA imaging showed eyes with recurrent macular edema had a less intact perifoveal capillary ring and a broader area of ring loss.^24^ CVs as well as edema may overshadow smaller NPAs on FA, limiting the validity of NPA-detection to larger areas, as small NPAs are harder to distinguish from surrounding tissue.^25^ In all eyes where GVs were identified on UWF-CF images, adjacent NPAs were also observed on WF-OCTA in both BRVO and CRVO, consistent with previous reports.^26^ Whereas Lip et al. described GV formation as a later manifestation of the disease, we did not detect a significant correlation between disease duration and the presence of GVs.^26^ It should be noted, however, that eyes with a disease duration of less than 12 weeks were not included in this study, which may represent the critical timeframe for GV development. In eyes with BRVO, CS were found significantly more often with NPAs present, supporting the hypothesis that arteries overlying and compressing retinal veins at arteriovenous crossings represent the site of retinal venous occlusion.^27^

In our cohort, two eyes showed NV formation (see Fig. 2). Before the advent of anti-VEGF drugs, ocular NVs appeared at a much larger rate. Hayreh et al. followed the natural course of 912 CRVOs and 190 hemi-CRVOs and found an NVE rate of 12% and 29% and an NVD rate of 10% and 12% for ischemic CRVOs and hemi-CRVOs, respectively.^28^ The SCORE study report #11 provided a 36-month incidence of NV in 8.8% and 7.6% for CRVO and BRVO, respectively.^29^ It is known that VEGF is the driving factor for ocular neovascularization.^30^ Therefore, the low number of eyes with NVs in our cohort (2.5%) may be due to the availability and proximity of free medical care in Austria, as affected patients usually have no trouble receiving treatment. A correlation between FAZ size and BCVA in RVO has previously been described.^31^^,^^32^ However, it is important to showcase that high quality images of the foveal region are possible even when acquiring images with a large FOV.

Glacet-Bernard et al. stitched five 12 × 12 mm PlexElite images together to create an FOV of approximately 60 degrees.^25^ The WF-OCTA prototype is equipped with a 1.680 MHz laser, resulting in high resolution images on a large FOV of 65 degrees (roughly 18 × 18 mm) while maintaining a fast image acquisition time of less than 15 seconds. This is especially important for patients with severe retinal nonperfusion and subsequent vision loss, as keeping the focus on a central point may get increasingly difficult with longer acquisition times. Glacet-Bernard et al. describe the long acquisition time as one of the main disadvantages of WF-OCTA resulting in motion artifacts and poor image quality, so eliminating this factor by achieving a 65-degree FOV within 15 seconds will further enhance the importance of WF-OCTA in clinical practice.^25^ Subsequently, this allowed for a detailed description of both central and peripheral retinal changes in RVO and their precise allocation in the retina to different subfields (see Fig. 4).

### Limitations

Despite the short image acquisition time of 15 seconds for an 18 × 18 mm grid, patients were still required to focus on a central point during acquisition. As stated above, of the 35 eyes that were excluded from this analysis, 4 eyes had to be excluded solely due to poor image quality, which may have led to selection bias as the lack of fixation may have been due to a more advanced disease stage. However, this was necessary to ensure correct measurement, because RVO is often accompanied by large subretinal hemorrhage overshadowing OCTA signals. One major limitation of this study is the lack of correction for axial length. Because PlexElite 12 × 12 images have been used as scale reference, NPA size values may not be correct and therefore, the 0.1 mm^2^ threshold for NPA annotation may include some NPA measurements that would otherwise be excluded and vice versa. Incorrectly scaled images may lead to incorrect measurements and NPA size values and therefore make size comparison to other studies challenging.^33^^–^^35^ However, we do believe that even if the size values measured in this analysis are just an approximation and therefore have to be treated as such, our results still hold value when evaluating associations between NPAs and other retinal pathologies. Another limitation is the measurement and comparability of SVD on images with larger FOV. It has previously been shown that with a wider FOV, smaller capillaries may not be captured when measuring SVD, therefore reducing the overall SVD values.^16^ Additionally, results may vary between devices. The presence of extensive NPAs may further lower SVD values. However, we believe that SVD measurements in this study underscore the presence of NPAs in the respective quadrant. Further, even though our WF-OCTA device offers a 65-degree FOV on single capture, it is not yet able to compete with the FOV of UWF-FA devices. However, as technological developments in OCTA continue to advance toward ultra-wide fields of view, we anticipate that an even more comprehensive assessment of peripheral retinal vascular characteristics will be possible in the near future. Because FA was not routinely performed in this subanalysis, we do not have direct comparison to FA image findings. Nevertheless, as mentioned before, we do believe that WF-OCTA offers equal, if not more precise evaluation of the retina, especially if used in conjunction with UWF-CF imaging.^23^^,^^25^ With the increasing availability of WF-OCTA in clinics around the world, its efficacy in a real-world data set is yet to be proven.

## Conclusions

We showed a detailed description of retinal findings using a combination of UWF-CF and WF-OCTA images. The formation of GVs seems to be a significant predictor for underlying retinal NPAs in both CRVO and BRVO, whereas presence of CVs seem to be a significant predictor for NPAs in eyes with BRVO. Additionally, for BRVO, IRF, IRH, and CS may be indicators for underlying NPAs. The combination of UWF-CF and WF-OCTA may provide important insights into retinal vascular alterations in clinical practice.

## Supplementary Material

Supplement 1
