# Supplementary material for: Retinal Characteristics in Eyes With Retinal Vein Occlusion Using Widefield Swept-Source Optical Coherence Tomography Angiography
Source: Invest Ophthalmol Vis Sci. 2026 Mar 19;67(3):45. doi: 10.1167/iovs.67.3.45 (PMC13012192; doi:10.1167/iovs.67.3.45)
Supplement: Supplement 1 [file iovs-67-3-45_s001.pdf]

Cohort	Variable	Spearman rho	Spearman p
**Overall**	disease duration vs NPA size	-0.09	0.436
	disease duration vs BCVA	-0.12	0.294
	disease duration vs CV	0.13	0.23
	disease duration vs MA	-0.03	0.794
	disease duration vs GV	0.09	0.438
	disease duration vs IRH	-0.17	0.138
	disease duration vs HE	0.06	0.612
	disease duration vs CW-spots	-0.38	**<0.001**
	disease duration vs anti-VEGF	0.71	**<0.001**
	anti-VEGF vs NPA size	-0.10	0.391
	anti-VEGF vs CV	0.08	0.501
	anti-VEGF vs MA	0.00	0.987
	anti-VEGF vs GV	0.02	0.834
	anti-VEGF vs IRH	-0.13	0.255
	anti-VEGF vs HE	0.14	0.225
	anti-VEGF vs CW-spots	-0.30	**0.007**
**BRVO**	disease duration vs NPA size	-0.18	0.225
	disease duration vs BCVA	-0.1	0-512
	disease duration vs CV	0.18	0.212
	disease duration vs MA	-0.12	0.426
	disease duration vs GV	0.05	0.726
	disease duration vs IRH	-0.24	0.104
	disease duration vs HE	-0.09	0.545
	disease duration vs CW-spots	-0.51	**<0.001**
	disease duration vs anti-VEGF	0.68	**<0.001**
	anti-VEGF vs NPA size	-0.15	0.304
	anti-VEGF vs CV	0.16	0.282
	anti-VEGF vs MA	-0.04	0.783
	anti-VEGF vs GV	0.08	0.603
	anti-VEGF vs IRH	-0.22	0.127
	anti-VEGF vs HE	0.14	0.354
	anti-VEGF vs CW-spots	-0.42	**0.003**
**CRVO**	disease duration vs NPA size	0.07	0.704
	disease duration vs BCVA	-0.05	0.797
	disease duration vs CV	0.06	0.723
	disease duration vs MA	0.1	0.589
	disease duration vs GV	0.24	0.179
	disease duration vs IRH	-0.06	0.721
	disease duration vs HE	0.29	0.102
	disease duration vs CW-spots	-0.10	0.563
	disease duration vs anti-VEGF	0.76	**<0.001**
	anti-VEGF vs NPA size	-0.03	0.874
	anti-VEGF vs CV	0.03	0.881
	anti-VEGF vs MA	0.00	0.999
	anti-VEGF vs GV	0.02	0.896
	anti-VEGF vs IRH	-0.07	0.689
	anti-VEGF vs HE	0.17	0.337
	anti-VEGF vs CW-spots	-0.01	0.911

Supplementary Table 1: Spearman correlations of disease duration with vascular and functional measures. Anti-VEGF = number of anti-vascular endothelial growth factor injections, BCVA = best corrected visual acuity, BRVO = branch retinal vein occlusion, CRVO = central retinal vein occlusion, CV = collateral vessels, CW-spots = cotton wool-spots, GV = ghost vessels, HE = hard exudates, IRH = intraretinal hemorrhage, MA = microaneurysms, NPA size = non-perfusion area size

		Univariate model		Model adjusted for duration	
**Cohort**	**Term**	**Coefficient (95% CI)**	**p**	**Coefficient (95% CI)**	**p**
**Overall**	Intercept	1.858 (1.41 - 2.306)	**<0.001**	2.092 (1.504 - 2.679)	**<0.001**
	GV	0.371 (0.206 - 0.535)	**<0.001**	0.371 (0.207 - 0.535)	**<0.001**
	Disease duration			-0.001 (-0.004 - 0.001)	0.227
**BRVO**	Intercept	1.642 (1.106 - 2.178)	**<0.001**	1.947 (1.277 - 2.616)	**<0.001**
	GV	0.362 (0.2 - 0.524)	**<0.001**	0.354 (0.194 - 0.515)	**<0.001**
	Disease duration			-0.002 (-0.004 - 0.001)	0.141
**CRVO**	Intercept	2.036 (1.239 - 2.833)	**<0.001**	2.143 (0.871 - 3.416)	**0.002**
	GV	0.653 (0.102 - 1.204)	**0.022**	0.673 (0.082 - 1.264)	**0.027**
	Disease duration			-0.001 (-0.008 - 0.007)	0.825

Supplementary Table 2: Linear regression models for log (1+non perfusion area size) versus ghost vessels (GV): univariable model and model adjusted for disease duration (in weeks). Results are shown for the overall cohort, branch retinal vein occlusion (BRVO) and central retinal vein occlusion (CRVO).

Outcome ~ Covariate	Cohort	Term	Coefficient (95% CI)	p
**Log (1+NPA size) ~ GV**	Overall	Intercept	1.72 (1.12 - 2.33)	**<0.001**
		GV	0.37 (0.2 - 0.55)	**<0.001**
		aHT	0.09 (-0.72 - 0.9)	0.827
		DM	0.13 (-1.1 - 1.35)	0.84
		CVD	0.55 (-0.68 - 1.78)	0.375
	BRVO	Intercept	1.64 (0.91 - 2.36)	**<0.001**
		GV	0.38 (0.21 - 0.56)	**<0.001**
		aHT	0.11 (-0.84 - 1.06)	0.813
		DM	-0.56 (-2.08 - 0.97)	0.467
		CVD	-0.2 (-2.15 - 1.76)	0.841
	CRVO	Intercept	2.03 (0.9 - 3.15)	**<0.001**
		GV	0.82 (0.11 - 1.52)	**0.025**
		aHT	-0.78 (-2.53 - 0.96)	0.364
		DM	0.46 (-1.77 - 2.7)	0.674
		CVD	1.17 (-0.79 - 3.14)	0.231
**Log (1+NPA size) ~ CV**	Overall	Intercept	1.68 (1.03 - 2.32)	**<0.001**
		CV	0.16 (0.07 - 0.25)	**<0.001**
		aHT	0.04 (-0.79 - 0.88)	0.918
		DM	1.14 (--0.07 - 2.34)	0.065
		CVD	-0.01 (-1.26 - 1.25)	0.99
	BRVO	Intercept	1.71 (0.87 - 2.56	**<0.001**
		CV	0.18 (0.03 - 0.32)	**0.017**
		aHT	-0.19 (-1.29 - 0.91)	0.731
		DM	0.6 (-1.01 - 2.21)	0.456
		CVD	-0.19 (-2.39 - 2)	0.86
	CRVO	Intercept	1.67 (0.51 - 2.83)	**0.007**
		CV	0.16 (0.03 - 0.29)	**0.017**
		aHT	0.15 (-1.36 - 1.67)	0.838
		DM	1.69 (-0.41 - 3.8)	0.111
		CVD	-0.08 (-1.9 - 1.74)	0.927
**FAZ size ~ BCVA**	Overall	Intercept	1.59 (1 - 2.18)	**<0.001**
		BCVA	-1.39 (-2.15 - -0.64)	**<0.001**
		aHT	-0.35 (-0.83 - 0.13)	0.153
		DM	-0.25 (-0.96 - 0.46)	0.489
		CVD	0.71 (-0.03 - 1.44)	0.06
	BRVO	Intercept	0.61 (0.42 - 0.81)	**<0.001**
		BCVA	-0.21 (-0.48 - 0.06)	0.117
		aHT	-0.08 (-0.23 - 0.07)	0.303
		DM	0.13 (-0.11 - 0.36)	0.285
		CVD	-0.06 (-0.37 - 0.25)	0.689
	CRVO	Intercept	2.77 (1.45 - 4.08)	**<0.001**
		BCVA	-2.66 (-4.38 - -0.94)	**0.004**
		aHT	-1.23 (-2.4 - -0.05)	**0.041**
		DM	-0.24 (-1.81 - 1.33)	0.76
		CVD	0.88 (-0.51 - 2.26)	0.207

Supplementary Table 3: Adjusted linear regression models analyzing the association of NPA and GV, CV as well as FAZ size and BCVA. All coefficients from the multivariable models are shown with 95% confidence intervals and p-values. aHT = arterial hypertension, BCVA = best corrected visual acuity, BRVO = branch retinal vein occlusion, CRVO = central retinal vein occlusion, CV = collateral vessels, CVD = cardiovascular disease, DM = diabetes mellitus, FAZ size = size of the foveal avascular zone, GV = ghost vessels

Cohort	Model	Term	Coefficient (95% CI)	P
**Overall**	log(1+NPA) ~ Location *CV	Intercept	1.990 (1.524 - 2.457)	**<0.001**
		CV >6-18mm	-0.1 (-0.77 - 0.57)	0.768
		covariate CV 0-6mm and CV >6-18mm	0.303 (0.116 - 0.49)	**0.002**
		CV >6-18mm : covariate CV 0-6mm and CV >6-18mm	-0.023 (-0.264 - 0.218)	0.851
	log(1+NPA) ~ CV optic disc	Intercept	2.35 (1.876 - 2.824)	**<0.001**
		CV optic disc	0.02 (-0.501 - 0.542)	0.938
**BRVO**	log(1+NPA) ~ Location *CV	Intercept	1.836 (1.22 - 2.453)	**<0.001**
		CV >6-18mm	-0.126 (-1.049 - 0.797)	0.786
		covariate CV 0-6mm and CV >6-18mm	0.305 (0.06 - 0.55)	**0.015**
		CV >6-18mm : covariate CV 0-6mm and CV >6-18mm	0.014 (-0.346 - 0.374)	0.938
	log(1+NPA) ~ CV optic disc	Intercept	2.318 (1.764 - 2.872)	**<0.001**
		CV optic disc	-0.796 (-2.512 - 0.92)	0.355
**CRVO**	log(1+NPA) ~ Location *CV	Intercept	2.193 (1.439 - 2.948)	**<0.001**
		CV >6-18mm	-0.118 (-1.193 - 0.958)	0.828
		covariate CV 0-6mm and CV >6-18mm	0.322 (0.017 - 0.627)	**0.039**
		CV >6-18mm : covariate CV 0-6mm and CV >6-18mm	-0.056 (-0.421 - 0.308)	0.758
	log(1+NPA) ~ CV optic disc	Intercept	2.45 (1.531 - 3.37)	**<0.001**
		CV optic disc	0.049 (-0.622 - 0.72)	0.883

Supplementary Table 4: Regression models with all coefficients analyzing the association of collateral vessels (CV) located around the posterior pole (0-6mm), peripheral retinal (>6-18mm) as well as on the optic disc and non-perfusion areas. Results are shown for the overall cohort, branch retinal vein occlusion (BRVO) and central retinal vein occlusion (CRVO), including coefficients with 95% confidence intervals and p-values.
